# Complex Spiking Neural Network Evaluated by Injury Resistance Under Stochastic Attacks

**DOI:** 10.3390/brainsci15020186

**Published:** 2025-02-13

**Authors:** Lei Guo, Chongming Li, Huan Liu, Yihua Song

**Affiliations:** 1 Tianjin Key Laboratory of Bioelectromagnetic Technology and Intelligent Health, School of Health Sciences and Biomedical Engineering, Hebei University of Technology, Tianjin 300131, China; 202232902013@stu.hebut.edu.cn (C.L.); 202011402002@stu.hebut.edu.cn (H.L.); 202212901005@stu.hebut.edu.cn (Y.S.); 2 State Key Laboratory of Reliability and Intelligence of Electrical Equipment, Hebei University of Technology, Tianjin 300131, China

**Keywords:** brain-inspired models, injury resistance, spiking neural network, complex network topology, synaptic plasticity, injury-resistance mechanism

## Abstract

Background: Brain-inspired models are commonly employed for artificial intelligence. However, the complex environment can hinder the performance of electronic equipment. Therefore, enhancing the injury resistance of brain-inspired models is a crucial issue. Human brains have self-adaptive abilities under injury, so drawing on the advantages of the human brain to construct a brain-inspired model is intended to enhance its injury resistance. But current brain-inspired models still lack bio-plausibility, meaning they do not sufficiently draw on real neural systems’ structure or function. Methods: To address this challenge, this paper proposes the complex spiking neural network (Com-SNN) as a brain-inspired model, in which the topology is inspired by the topological characteristics of biological functional brain networks, the nodes are Izhikevich neuron models, and the edges are synaptic plasticity models with time delay co-regulated by excitatory synapses and inhibitory synapses. To evaluate the injury resistance of the Com-SNN, two injury-resistance metrics are investigated and compared with SNNs with alternative topologies under the stochastic removal of neuron models to simulate the consequence of stochastic attacks. In addition, the injury-resistance mechanism of brain-inspired models remains unclear, and revealing the mechanism is crucial for understanding the development of SNNs with injury resistance. To address this challenge, this paper analyzes the synaptic plasticity dynamic regulation and dynamic topological characteristics of the Com-SNN under stochastic attacks. Results: The experimental results indicate that the injury resistance of the Com-SNN is superior to that of other SNNs, demonstrating that our results can help improve the injury resistance of SNNs. Conclusions: Our results imply that synaptic plasticity is an intrinsic element impacting injury resistance, and that network topology is another element that impacts injury resistance.

## 1. Introduction

Brain-inspired models have been commonly employed for artificial intelligence [1,2,3]. With the development of industrialization and informatization, the complex environment has become more complicated. The interference can injure electronic components, resulting in reduced functioning or, possibly, complete breakdown in the system [4,5], meaning that enhancing the injury resistance of brain-inspired models is a crucial issue. Through continuous refinement by natural selection, human brains have self-adaptive abilities under injury [6,7]. Drawing on the self-adaptivity of human brains, the construction of a bio-plausible brain-inspired model is intended to enhance its injury resistance. However, current brain-inspired models lack bio-plausibility, meaning they do not sufficiently draw on real neural systems’ structure or function. The spiking neural network (SNN) is a brain-inspired model that faithfully draws on biological electric characteristics to provide information-processing abilities [8,9,10]. Currently, SNNs have achieved good performance in pattern recognition tasks such as speech recognition [11], image recognition [12], and object tracking [13]. This type of structure is made by a neuron model, a synaptic plasticity model, and topology.

Bio-neurons serve as neuronal information-processing units in brain, and neuron models are computational models that simulate the electrophysiological properties of bio-neurons [14]. The Hodgkin–Huxley neuron model, a fourth-order ordinary differential equation, accurately explains the connection between the neuronal membrane potential (MP) and the ion channel currents but has a high computing cost [15]. Conversely, the leaky integrate-and-fire neuron model, a first-order ordinary differential equation, reduces the computational cost, but cannot conform well to the firing properties of bio-neurons [16]. The Izhikevich neuron model, a second-order ordinary differential equation, balances the need to conform closely to the spiking properties of bio-neurons with a relatively lower computing cost [17], and is broadly used in the construction of SNNs. In a previous study [18], we built a scale-free spiking neural network (SF-SNN) using the Izhikevich neuron model and examined the neural coding of the SF-SNN for different exterior noises. Our results indicated that the SF-SNN can distinguish between types of noise based on specific neural coding.

As connections between neurons, synapses make up the neurological foundation of learning and information transfer [19]. According to biological studies, inhibitory synapses (ISs) are significant because they establish dynamic adjustment in a bio-brain with excitatory synapses (ESs) [20]. Depending on findings for bio-synapses, researchers have modeled synaptic plasticity co-regulated by ESs and ISs to construct SNNs. For example, Zhao et al. [21] found that the SNN with ESs and ISs was better than the SNN without ISs on the MNIST image datasets. For bio-synapses, neurotransmitter dispersion causes a synaptic time delay (STD), which enhances the ability of information processing and is stochastically dispersed between 0.1 and 40 ms [22]. Zhang et al. [23] constructed an SNN with an STD and found that the learning accuracy of this SNN was better than that of an SNN without an STD in producing the target pattern of the sequence of spikes. Thus, synaptic plasticity models with an STD consistent with a bio-STD, which is co-regulated by ESs and ISs, can enhance the bio-plausibility of the SNN.

The topology decides how neurons are connected and impacts the function of the network. Van et al. [24] used fMRI data to construct functional brain networks (FBNs), and the computational findings in regard to graph characteristics indicated that these human brain networks had scale-free (SF) and small-word (SW) properties. To simulate the topology of human brains, researchers have generated topologies with SF and/or SW properties using algorithms to construct SNNs. Hu et al. [25] used the Barabási–Albert (BA) algorithm to construct an SF-SNN and found that there was an optimal maximum weight value for the spike-timing-dependent plasticity in terms of enhancing the synchronization of the SF-SNN. Tsakalos et al. [26] used the Watts and Strogatz (WS) algorithm to construct a small-world spiking neural network (SW-SNN) and discovered that its recognition accuracy was higher than that of a two-layered SNN on the MNIST image dataset. According to the topological characteristics of biological FBNs, the complex SNN (Com-SNN) with both SF and SW properties can enhance the bio-plausibility of the SNN.

The human brain exhibits self-adaptability to exterior stimulation [6]. Drawing on the self-adaptability of human brains, researchers have investigated the responses of brain-inspired models to exterior stimulation and have found that they show self-adaptively regulatory behaviors, such as synchrony [27] and resonance [28]. The disturbance resistance and injury resistance of brain-inspired models is another aspect of self-adaptive adjustment [29]. In a previous study [30], we investigated the disturbance resistance of an SW-SNN and SF-SNN and found that the SW-SNN was superior to the SF-SNN under impulse noise. Jang et al. [31] examined the injury resistance of an NN with various activation functions and discovered that this network’s injury resistance is better than that of an NN without various activation functions. Based on the results of biological experiments [32], the human brain connection is relatively resistant to an attack againstnodes compared to SF networks. Thus, a more bio-plausible topology would improve the injury resistance of brain-like models. However, current brain-inspired models still lack bio-plausibility, and the injury-resistance mechanism of brain-inspired models remains unclear.

To address the above challenges, the purpose of this paper is to construct a brain-inspired model with bio-plausibility drawing on the advantages of human brain structures, in an attempt to enhance its injury resistance. In addition, this paper analyzes the injury-resistance mechanism of the Com-SNN by synaptic plasticity dynamic regulation (SPDR) and dynamic topological characteristics (DTC) under stochastic attacks.

The main contributions of this paper are as follows:

- To improve the bio-plausibility of brain-inspired models, a new SNN is presented called Com-SNN, in which the topology is inspired by the topological characteristics of FBNs, the nodes are Izhikevich neuron models, and the edges are synaptic plasticity models with an STD co-regulated by ESs and ISs.
- To evaluate the injury resistance of the Com-SNN, two injury-resistance metrics are investigated, and it is found that the injury resistance of the Com-SNN is superior to that of SNNs with alternative topologies under the stochastic removal of neuron models to simulate the consequence of stochastic attacks.
- To reveal the injury-resistance mechanism of the Com-SNN, the SPDR and DTC under stochastic attacks are analyzed. The results imply that synaptic plasticity is an intrinsic element impacting injury resistance, and that network topology is another element that impacts injury resistance.

The remainder of this study is arranged as follows. The method of constructing the Com-SNN is presented in Section 2. The evaluation of the injury resistance and injury-resistance mechanism of the Com-SNN is presented in Section 3. The discussion is presented in Section 4. Finally, a conclusion is presented in Section 5.

## 2. Materials and Methods

We describe the construction of SNNs. To enhance the bio-plausibility of SNNs, the Com-SNN is constructed. To compare the impact of the topology on the injury resistance of the SNN, SNNs with alternative topologies (SW-SNN and SF-SNN) are constructed.

### 2.1. Construction of Com-SNN

In our Com-SNN model, we introduce the Barrat–Barthelemy–Vespignani (BBV) method to produce the topology, incorporating Izhikevich neuron models as nodes, and synaptic plasticity models with an STD co-regulated by ESs and ISs as edges.

#### 2.1.1. Topology of Com-SNN

We generate a network with SF and SW properties inspired by the topological characteristics of FBNs to obtain the topology of the Com-SNN. The BBV algorithm is used to produce networks with SF and SW properties. The BBV algorithm is able to produce alternative topologies of networks through adapting reattachment possibility factor $p_{n}$ [33]. By analyzing the SF and SW characteristics, a suitable $p_{n}$ needs to be chosen to produce a bio-plausible network.

To analyze the network properties of Com-SNNs, we computed the SF property γ [34] and SW property σ [35] for networks with 0.1 to 0.9 with 0.1/step for $p_{n}$. The network’s scale is 500 nodes. Table 1 shows the γ and σ for different $p_{n}$.

**Figure 1:**
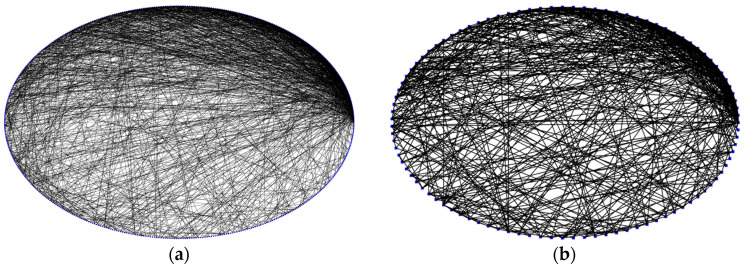
Topological visualization of Com-SNN: (**a**) Com-SNN with 500 nodes; (**b**) Com-SNN with 100 nodes. (Blue dots represent nodes and black lines represent edges).

From Table 1, it can be seen that when $p_{n}=0.3$, γ=2.12 is the closest to the SFP of the FBN, which is about 2 [36]; when $p_{n}=0.3$, σ=1.96 is the largest, which is in line with the range of the SWP of the FBN [37]. Therefore, we choose $p_{n}=0.3$ for generating the topology of the Com-SNN. In Figure 1a, its topology is shown. To enhance the clarity of the Com-SNN topology illustration, we decrease the number of nodes in Figure 1b from 500 to 100.

The topology we constructed combines the SF and SW properties and conforms to the range of properties of FBNs.

#### 2.1.2. Izhikevich Neuron Model

**Figure 2:**
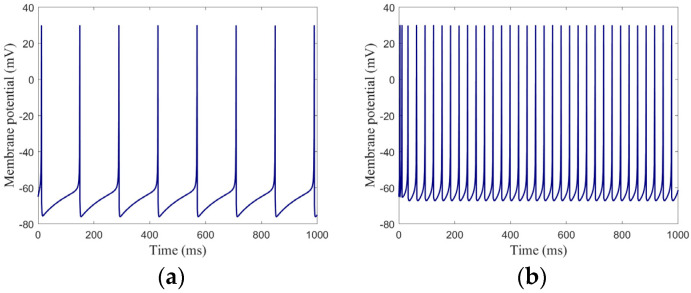
Firing sequences: (**a**) excitatory neuron; (**b**) inhibitory neuron.

According to the description in the introduction, the Izhikevich neuron model balances the need to conform closely to the spiking properties of bio-neurons with a relatively lower computing cost [17] compared to the Hodgkin–Huxley neuron model [15] and leaky integrate-and-fire neuron model [16], and is broadly used in the construction of SNNs. Hence, we use the Izhikevich neuron model to represent the nodes of the Com-SNN as follows [17]:

(1) $$\left\{\begin{array}{l} dv/dt=0.04v^{2}+5v+140-u+I_{ext}+I_{g}\\ du/dt=a\left(bv-u\right)\\ if\;v\geq 30,\;then\left\{\begin{array}{l} v\leftarrow c\\ u\leftarrow u+d \end{array}\right. \end{array}\right.$$

where v denotes the MP, u denotes the recovery variable of v, $I_{ext}$ denotes the external current, and $\;I_{g}\;$ denotes the synaptic current. a, b, c, and d are four non-dimensional parameters that can be adjusted to simulate various neuron firing sequences. Figure 2 shows the firing sequences.

In this study, regular spiking was used as the firing pattern of excitatory neurons in the neural network as shown in Figure 2a; low-threshold spiking was used as the firing pattern of inhibitory neurons as shown in Figure 2b. These firing sequences demonstrate how the Izhikevich model effectively captures biologically realistic neuron dynamics.

#### 2.1.3. Synaptic Plasticity Model

The synaptic plasticity models we constructed introduce a stochastic time delay consistent with a bio-STD and the co-regulation of ESs and ISs.

An STD is introduced to denote the diffusion of neurotransmitters in bio-synapses and expressed as follows [38]:

(2) I_syn(t)=g(t)r_g(t)(E_syn−V_post(t))

where $I_{syn}$ is the synaptic current, $V_{post}$ denotes the postsynaptic MP, and g denotes the synaptic weight. $r_{g}$ denotes the reversible potential H, as follows:

(3) $$\left\{\begin{array}{c} dr_{g}/dt=\alpha H\left(1-r_{g}\right)-\beta r_{g}\\ H={\left[1+\exp\left(-V_{pre}(t-\tau )\right)\right]}^{-1} \end{array}\right.$$

where $V_{pre}$ represents the presynaptic MP. The ES weight $g_{ex}$ and IS weight $g_{in}$ are regulated as follows [39].

When a postsynaptic neuron j does not receive an action potential from a presynaptic neuron i, the $g_{ex}$ and $g_{in}$ slump as follows:

(4) $$\left\{\begin{array}{c} \mu_{ex}\frac{dg_{ex}}{dt}=-g_{ex}\\ \mu_{in}\frac{dg_{in}}{dt}=-g_{in} \end{array}\right.$$

When j receives action from i, $g_{ex}$ and $g_{in}$ change as follows:

(5) $$\left\{\begin{array}{l} g_{ex}\left(t\right)=g_{ex}\left(t\right)+{\bar{g}}_{ex}\\ {\bar{g}}_{ex}=w\left(∆t\right)\times g_{max} \end{array}\right.$$

(6) $$\left\{\begin{array}{l} g_{\mathrm{in}}\left(t\right)=g_{in}\left(t\right)+{\bar{g}}_{in}\\ {\bar{g}}_{in}=m\left(∆t\right)\times g_{max} \end{array}\right.$$

where ${\bar{g}}_{ex}$ and ${\bar{g}}_{in}$ represent the excitatory and inhibitory conductance increments induced by the action potential, respectively. $g_{max}$ is the max synaptic weight; if ${g>g}_{max}$, it is set to $g_{max}$, whereas if $g<g_{max}$, it is set to 0. The excitatory synaptic weight increment ${\bar{g}}_{ex}$ is regulated by w(∆t), and the inhibitory synaptic weight increment ${\bar{g}}_{in}$ is regulated by m(∆t). w(∆t) and m(∆t) are related to the impulse discharges of the presynaptic neuron and the postsynaptic neuron, which can be expressed as follows:

(7) $$w\left(∆t\right)=\left\{\begin{array}{l} A_{+}\exp(\Delta t/t_{+}),\Delta t<0\\ -A_{-}\exp(-\Delta t/t_{-}),\Delta t\geq 0 \end{array}\right.$$

(8) $$m\left(∆t\right)=\left\{\begin{array}{l} -B_{+}\exp(\Delta t/t_{+}),\Delta t<0\\ B_{-}\exp(-\Delta t/t_{-}),\Delta t\geq 0 \end{array}\right.$$

where ∆t is the interval between the time of the presynaptic spike and the time of the postsynaptic spike. $t_{+}$ and $t_{-}$ are the intervals between pre- and postsynaptic neuron discharges during synaptic enhancement and weakening. $A_{+}$ and $A_{-}$ are the maximum corrections of synaptic conductance during the enhancement and weakening of excitation; $B_{+}$ and $B_{-}$ are the maximum corrections of synaptic conductance during the enhancement and weakening of inhibition.

### 2.2. Construction of SNNs with Alternative Topologies

To investigate whether topology impacts the injury resistance of an SNN, SNNs with two alternative topologies (SF-SNN and SW-SNN) were constructed. The network size, neuron model, and synaptic plasticity model were identical to those in the Com-SNN, and the only difference lay in the topology. The SF-SNN and SW-SNN were generated as follows.

#### 2.2.1. SF-SNN

**Figure 3:**
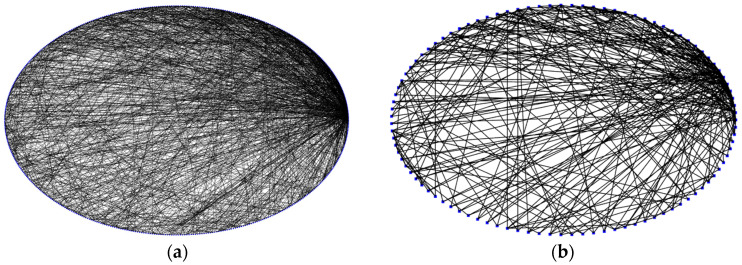
Topological visualization of SF-SNN: (**a**) SF-SNN with 500 nodes; (**b**) SF-SNN with 100 nodes. (Blue dots represent nodes and black lines represent edges).

The following stages are included in the BA algorithm [25], which produced the topology of the SF-SNN. (1) A network with $m_{0}$ nodes that are fully linked is created. (2) The network gains a new node with $m_{e}$ edges, where $m_{e}$ is the number of edges of previous nodes. Different γ may be used to build SF topologies by varying the network parameters $m_{0}$ and $m_{e}$. We adjusted the network parameters $m_{0}$ and $m_{e}$ to construct an SF-SNN with a value of γ = 2.12, which is also the value for the Com-SNN, to facilitate comparison. In Figure 3a, its topology is displayed. To enhance the clarity of the SF-SNN topology illustration, we decrease the number of nodes in Figure 3b from 500 to 100.

#### 2.2.2. SW-SNN

**Figure 4:**
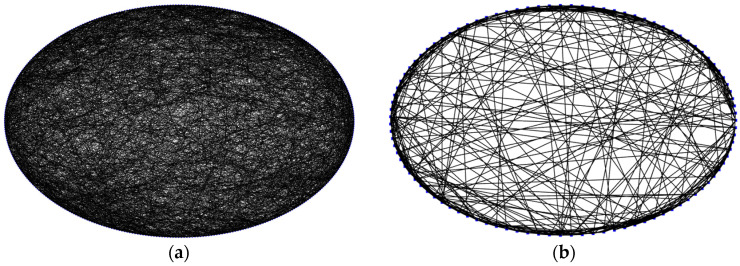
Topological visualization of SW-SNN: (**a**) SW-SNN with 500 nodes; (**b**) SW-SNN with 100 nodes. (Blue dots represent nodes and black lines represent edges).

The following stages are included in the WS algorithm [26], which produced the topology of the SW-SNN. (1) N nodes are created in a regular ring network, with each linked to its K closest neighbors. (2) Based on a reconnection probability, each edge is linked at random. Different values of σ may be obtained for SW topologies by manipulating the reconnection probability values. In order to facilitate comparison, we adjusted the reconnection probability to construct an SW-SNN with the same value, σ = 1.96, as the Com-SNN. In Figure 4a, its topology is shown. To enhance the clarity of the SW-SNN topology illustration, we decrease the number of nodes in Figure 4b from 500 to 100.

## 3. Results

In this section, we first introduce the experimental settings used in our experiments. Then, to evaluate the injury resistance of the Com-SNN, two injury-resistance metrics are investigated and compared with SNNs with alternative topologies under stochastic attacks.

### 3.1. Experimental Settings

In this section, we first introduce the experimental settings used in our experiments. Then, to evaluate the injury resistance of the Com-SNN, two injury-resistance metrics are investigated and compared with SNNs with alternative topologies under stochastic attacks.

#### 3.1.1. Izhikevich Neuron Model Parameters

According to [40], the Izhikevich neuron models, both excitatory and inhibitory, are dispersed stochastically within the SNNs in a 4:1 proportion. In accordance with the literature [41], Table 2 provides instructions and values for a, b, c, and d in excitatory and inhibitory neuron models.

#### 3.1.2. Synaptic Plasticity Model Parameters

According to biological research, the bio-STD is stochastically distributed in the interval of 0.1 to 40 ms [22]. Therefore, we incorporate an STD into the synaptic plasticity model following a stochastic distribution of bio-synaptic. The parameter instructions and values in the synaptic plasticity model, drawn from the literature [38,39], are shown in Table 3.

### 3.2. Comparison of the Injury Resistance of SNNs

To evaluate the injury resistance of the Com-SNN, two injury-resistance metrics are compared with SF-SNN and SW-SNN for 5% to 50% with 5%/step for the stochastic attacks proportion (P).

#### 3.2.1. Relative Change Rate in Firing Rate

The firing rate (FR) is the number of spikes released by a neuron per unit of time. The difference between the FR for the neuron model within the SNN under stochastic attacks and the FR for the matching neuron model without attacks is characterized by $\delta_{i}$. δ is the average of $\delta_{i}$, and can be expressed as follows:

(9) $$\delta =\sum_{i=1}^{N}\delta_{i}/N=\sum_{i=1}^{N}\frac{\left|f_{bi}-f_{ai}\right|}{f_{ai}}\times 100\%/N$$

where $f_{ai}$ is the FR of the neuron model within the SNN without attacks, and $f_{bi}$ is the FR of the corresponding neuron model in the SNN with attacks. δ is the mean of $\delta_{i}$, which denotes the change in neuron models between the SNN without attacks and the SNN with attacks. Hence, the lower δ, the better the injury resistance.

The results for δ of SNNs with three alternative topologies under stochastic attacks are compared in Table 4.

**Figure 5:**
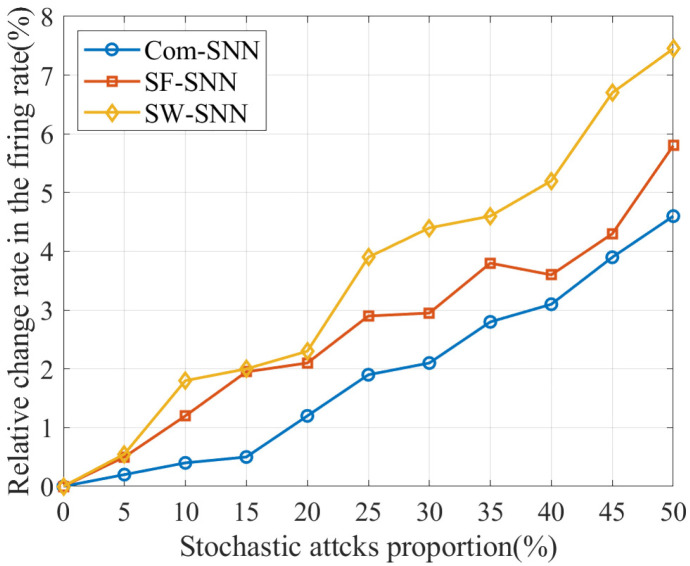
Results for δ of SNNs with alternative topologies under stochastic attacks.

In order to visualize the trend of δ of SNNs with three alternative topologies under stochastic attacks, we plotted the data in Table 4 as Figure 5.

As can be seen in Figure 5, δ for the Com-SNN is smaller than that for SNNs with alternative topologies under stochastic attacks, indicating that the injury resistance of the Com-SNN is superior to that of SNNs with alternative topologies on δ.

#### 3.2.2. Correlation Between Membrane Potentials

The correlation between the MP for a neuron model within an SNN under stochastic attacks and the MP for a corresponding neuron model without attacks is characterized by $\rho_{i}$. ρ is the average of the $\rho_{i}$ values, and can be expressed as follows:

(10) $$\rho =\sum_{i=1}^{N}\rho_{i}/N=\sum_{i=1}^{N}\frac{\sum_{t_{j}=t_{1}}^{t_{2}}x_{ai}\left(t_{j}\right)x_{bi}\left(t_{j}\right)}{\sqrt{\sum_{t_{j}=t_{1}}^{t_{2}}x_{ai}^{2}\left(t_{j}\right)\sum_{t_{j}=t_{1}}^{t_{2}}x_{bi}^{2}\left(t_{j}\right)}}/N$$

where $x_{ai}$ denotes the MP of a neuron model of the SNN before injury, reflecting its normal conditions. $x_{bi}$ denotes the MP of the corresponding neuron model after injury, reflecting its altered dynamics. The interval $\left[t_{1},t_{2}\right]$ denotes the time frame over which the correlation between the MPs is calculated. For our study, the total simulation duration is 1000 ms; we set $t_{1}=1\;ms$ and $t_{2}=1000\;ms$. N is the number of neuron models, which does not include those removed by stochastic attacks of the SNN. We evaluate the injury resistance of the SNN by measuring the mean of $\rho_{i}$, which denotes the average correlation between the MPs of the neuron models in the SNN before and after injury by stochastic attacks. A higher ρ indicates that neuronal activity remains stable under stochastic attacks, suggesting stronger injury resistance. Hence, the higher ρ, the better the injury resistance.

The results for the ρ of SNNs with three alternative topologies (Com-SNN, SF-SNN, and SW-SNN) under stochastic attacks are compared in Table 5.

**Figure 6:**
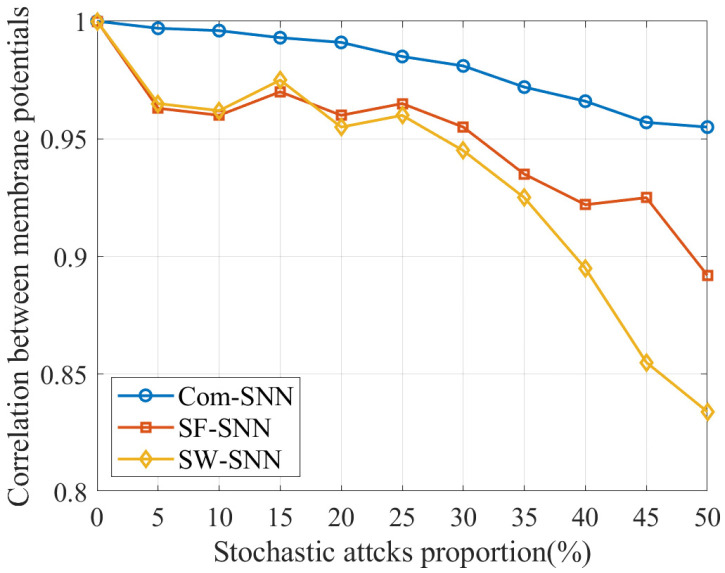
Results for ρ of SNNs with alternative topologies under stochastic attacks.

In order to visualize the trend of the ρ of SNNs with three alternative topologies under stochastic attacks, we plotted the data in Table 5 as Figure 6.

As can be seen in Figure 6, ρ for Com-SNN is larger than for SNNs with alternative topologies under stochastic attacks, indicating that the injury resistance of the Com-SNN is superior to that of SNNs with alternative topologies on ρ.

In summary, the injury resistance of the Com-SNN under stochastic attacks is superior to that of SNNs with alternative topologies, thus demonstrating that our approach enhances the injury resistance of SNNs.

### 3.3. Injury-Resistance Mechanism of Com-SNN

To reveal the injury-resistance mechanism of the Com-SNN, SPDR and DTC for our model under stochastic attacks are analyzed.

#### 3.3.1. Synaptic Plasticity Dynamic Regulation

To explore the reason why our Com-SNN model shows better injury resistance under stochastic attacks, we analyze the injury-resistance mechanism in terms of SPDR. To demonstrate the correlation between synaptic plasticity and injury-resistance metrics, we present the progression of mean synaptic weight (MSW) and progression of injury-resistance metrics of the Com-SNN under stochastic attacks; then, Pearson correlation analysis is used to examine their correlation.

1. Progression of mean synaptic weight

**Figure 7:**
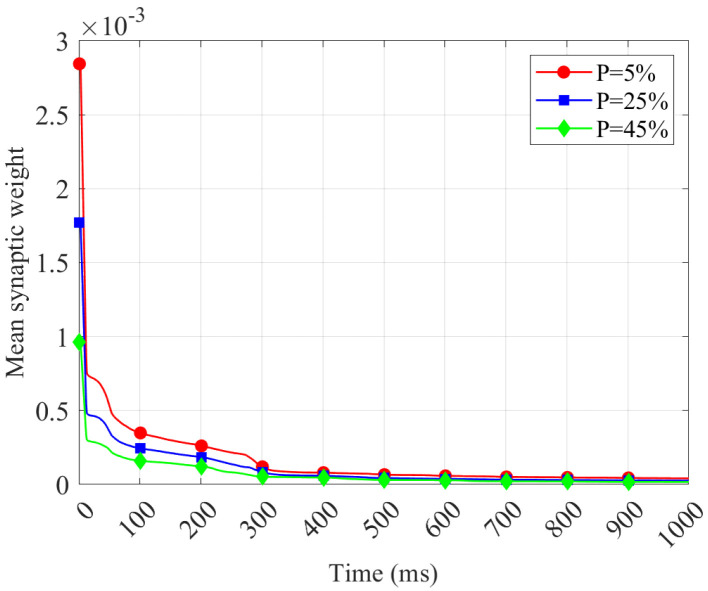
Progression of MSW for Com-SNN under stochastic attacks.

The MSW is the mean weight of all the synapses within the network. We calculate the progression of the MSW of the Com-SNN under three values of P (5%, 25%, and 45%) in Figure 7.

As can be seen in Figure 7, the progression of the MSWs for the Com-SNN under stochastic attacks shows comparable trends: In the first 100 ms, the MSWs decrease sharply, and then decrease slowly and progressively stabilize. This result reflects the process of SPDR under stochastic attacks.

2. Progression of injury-resistance metrics

**Figure 8:**
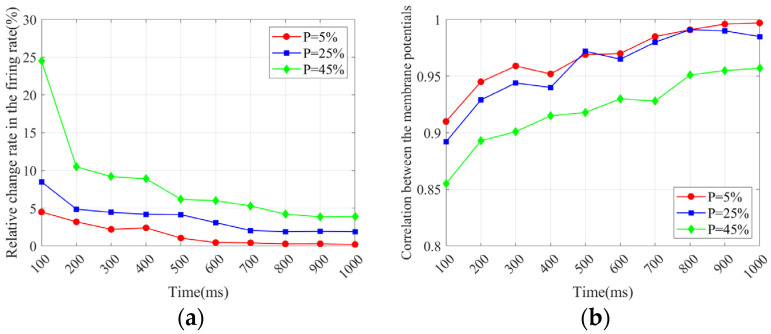
Progression of injury resistance of Com-SNN under stochastic attacks: (**a**) δ; (**b**) ρ.

We calculate the progression of the injury-resistance metrics (δ and ρ) of the Com-SNN under three values of P (5%, 25%, and 45%) in Figure 8.

As can be seen in Figure 8, the progression of the injury-resistance metrics of the Com-SNN under stochastic attacks exhibits comparable trends: Over the first 200 ms, these injury-resistance metrics change sharply, and then change slowly and progressively stabilize.

3. Relevance analysis

Pearson correlation analysis [42] is used for examining the impact of progression on the MSW and injury-resistance metrics under stochastic attacks. Additionally, a *t*-test [43] is performed to determine significant correlations; the 0.01 level significance is indicated by “**”. The relevance findings are shown in Table 6.

As can be seen from Table 6, the progression of the MSW shows significant correlation with δ and ρ at the 0.01 level. Our findings suggest that an intrinsic element impacting injury resistance is synaptic plasticity.

#### 3.3.2. Dynamic Topological Characteristics

To investigate the reason why there are different injury resistances of SNNs with the same synaptic plasticity but alternative topologies, we assess the mechanism in terms of the progression of the DTC.

1. Comparison of the mean weighted clustering coefficient for SNNs

We compare the mean weighted clustering coefficient (MWCC) for our Com-SNN with that of the SF-SNN and SW-SNN under stochastic attacks. The MWCC (${\tilde{C}}_{w}$) denotes the mean degree of agglomeration for all nodes within the SNN and reflects the efficiency of partial message transfer [44]. It is defined as follows:

(11) $${\tilde{C}}_{w}=\frac{1}{N}\sum_{i=1}^{N}\left(\frac{1}{s_{i}\left(k_{i}-1\right)}\sum_{j,k}\frac{\left(g_{ij}+g_{ik}\right)}{2}a_{ij}a_{jk}a_{ki}\right)$$

where $s_{i}$ is the node strength, $k_{i}$ is the node degree, $g_{ij}$ and $g_{ik}$ denote the synaptic weights, and $a_{ij}$, $a_{jk}$, and $a_{ki}$ denote the adjacent matrices.

**Figure 9:**
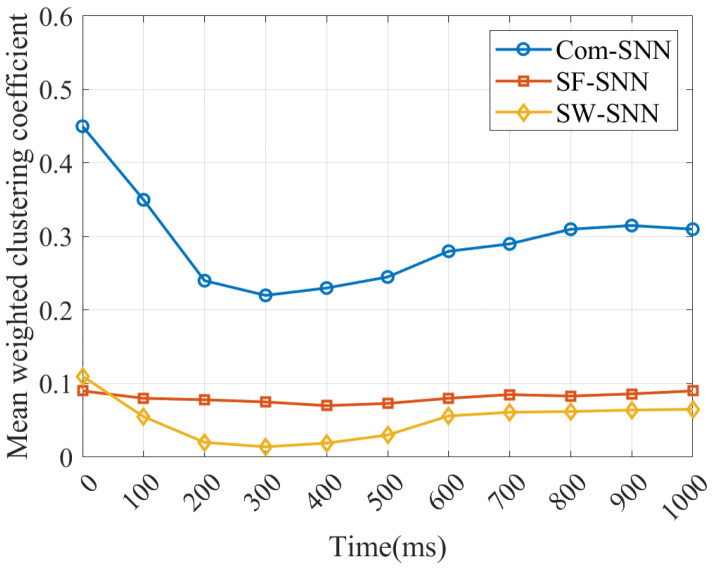
Progression of ${\tilde{C}}_{w}$ of SNNs under stochastic attacks.

In this study, the progression of ${\tilde{C}}_{w}$ for three SNNs with alternative topologies is compared under P=25% in Figure 9.

As can be seen in Figure 9, the ${\tilde{C}}_{w}$ for Com-SNN is larger than for SNNs with alternative topologies under stochastic attacks, indicating that the efficiency of partial message transfer of the Com-SNN is better.

2. Comparison of the mean weighted shortest path length for SNNs

We compare the mean weighted shortest path length (MWSPL) of our Com-SNN with that of SNNs with alternative topologies under stochastic attacks. The MWSPL (${\tilde{L}}_{w}$) denotes the mean of the shortest distances among node pairs within an SNN. This metric of the efficiency of global message transfer [44] is defined as follows:

(12) $${\tilde{L}}_{w}=\frac{1}{N(N-1)}\underset{ϒ(i,j)\in \Gamma (i,j)}{min}\left[\sum_{m,n\in ϒ(i,j)}\frac{1}{g_{mn}}\right]$$

where the synaptic weights are denoted by $g_{mn}$, the paths from neuron i to j are denoted by $\Upsilon \left(i,j\right)$, and the potential paths from neuron i to j are denoted by $\Gamma \left(i,j\right)$.

**Figure 10:**
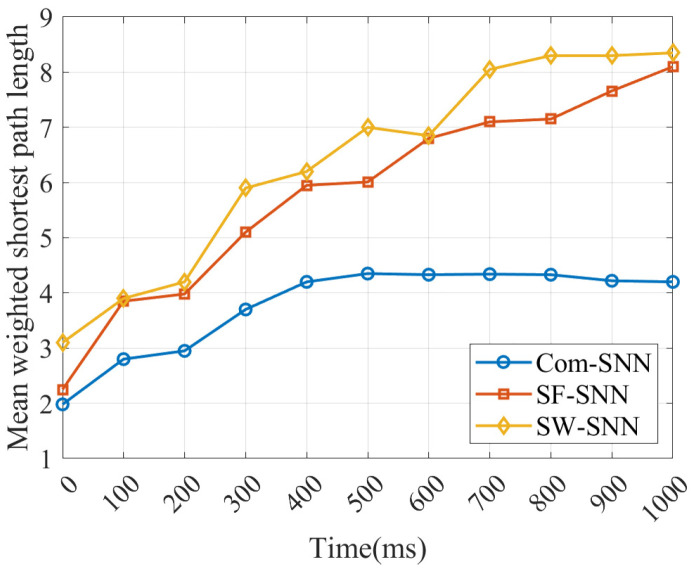
Progression of ${\tilde{L}}_{w}$ of SNNs under stochastic attacks.

In this study, the progression of the ${\tilde{L}}_{w}$ for SNNs with alternative topologies is compared under P=25% in Figure 10.

As can be seen in Figure 10, ${\tilde{L}}_{w}$ for Com-SNN is smaller than for SNNs with alternative topologies under stochastic attacks, indicating that efficiency of global message transfer of the Com-SNN is better.

In summary, these results show that efficiency of message transfer for the Com-SNN is higher than for the SF-SNN and SW-SNN, a finding that supports the results for injury resistance. Our results, therefore, suggest that topology is an element that impacts the injury resistance of SNNs.

These results imply that synaptic plasticity is an intrinsic element impacting injury resistance, and that network topology is another element that impacts injury resistance, and together, they influence adaptive neural restructuring under the influence of injury.

## 4. Discussion

In this section, we discuss comparisons with existing research, applications, and the limitations of the Com-SNN.

### 4.1. Comparisons with Existing Research

Our results offer important contributions to the field of brain-inspired models, particularly in terms of injury resistance. For topology, compared to previous studies [25,26], which focused on topologies with SF or SW properties, our Com-SNN model incorporates the topology inspired by the topological characteristics of biological functional brain networks that significantly improve injury resistance. For the synaptic plasticity model, compared to a previous study [23] focusing on fixed STD, our Com-SNN model incorporates synaptic plasticity models with time delay co-regulated by excitatory synapses and inhibitory synapses that significantly improve injury resistance. For injury resistance, compared to a previous study [30] focusing on the performance of SNNs under impulse noise, the Com-SNN represents a more adaptive approach that recovers from neuron loss, demonstrating superior robustness under stochastic attacks. These comparisons highlight the strengths of our work.

### 4.2. Applications

The Com-SNN has significant applications for neuromorphic hardware, especially in environments where robustness and adaptability are crucial. Its ability to maintain functionality despite neuron loss can enhance autonomous systems in unpredictable or injury environments. For example, in a pattern recognition system, where neuromorphic hardware failures or environmental injury can occur, the Com-SNN can ensure continued performance under injury. These applications connect theoretical insights from our work to the real-world challenges of injury resistance in brain-inspired models.

### 4.3. Limitations

While the Com-SNN model demonstrates promising results, several limitations need to be addressed. First, although the topology of the Com-SNN is consistent with the biological range, it still does not reflect the true connectivity of bio-brain networks. Another limitation is that while our model performs well in simulated environments, real-world implementations may encounter additional challenges related to noise, hardware constraints, and environmental variability. Finally, ethical considerations must be addressed when applying the Com-SNN in sensitive areas such as healthcare and autonomous systems. For instance, the use of brain–computer interfaces or neural prosthetics raises concerns about privacy, consent, and the potential for misuse. Additionally, deploying autonomous systems that rely on the Com-SNN must ensure that the technology is free from biases that could affect human safety.

## 5. Conclusions

In this study, we have proposed a brain-inspired model with bio-plausibility, called the Com-SNN, in which the topology is inspired by the topological characteristics of biological FBNs. To evaluate its injury resistance, two injury-resistance metrics of the Com-SNN under stochastic attacks are investigated, then compared with alternative-topology SNNs. Furthermore, the injury-resistance mechanism of the Com-SNN is analyzed. The following major findings may be taken from our work:

- The injury resistance of the Com-SNN under stochastic attacks is superior to that of SNNs with alternative topologies (SF-SNN and SW-SNN). Our results can help improve hte injury resistance of SNNs under stochastic attacks.
- Our results imply that synaptic plasticity is intrinsic element impacting injury resistance, and that network topology is another element that impacts injury resistance.

For future work, we aim to extend our model for implementation on neuromorphic hardware, which will allow us to assess its performance in more realistic and dynamic environments. Transitioning from simulation-based studies to neuromorphic systems presents several challenges. Firstly, we will need to address the scalability of our model to ensure it can operate efficiently on hardware with potentially limited computational resources. Additionally, the real-environment validation of injury resistance presents challenges such as introducing real-world noise and variability, which may introduce new forms of injury that our model needs to withstand. Through these future steps, we hope to bridge the gap between the Com-SNN and practical implementations, allowing for broader applications in neuromorphic hardware.

## Figures and Tables

**Table 1:** The γ and σ of for different $p_{n}$.

$p_{n}$	0.1	0.2	0.3	0.4	0.5	0.6	0.7	0.8	0.9
γ	1.59	1.78	2.12	2.29	2.52	2.80	2.88	2.93	3.11
σ	1.31	1.44	1.96	1.75	1.69	1.66	1.60	1.51	1.45

**Table 2:** Parameters of Izhikevich neuron models.

Parameter	Instruction	Value
a	Timescale for u	Excitement: 0.02
		Inhibition: 0.02
b	Sensitivity of u to sub-threshold fluctuations in v	Excitement: 0.20
		Inhibition: 0.25
c	Reset value of v	Excitement: −65
		Inhibition: −65
d	Reset value of u	Excitement: 8
		Inhibition: 2

**Table 3:** Parameters of the synaptic plasticity model.

Parameter	Instruction	Value
E [38]	Reversible synaptic potential	Excitement: 0 mV
		Inhibition: −70 mV
α [38]	Forward rate constant of the neurotransmitter	Excitement: 2
		Inhibition: 0.9
β [38]	Reverse rate constant of the neurotransmitter	Excitement: 1
		Inhibition: 0.1
$\mu_{ex}$ [39]	Decay constant of the ES weight	3 ms
$\mu_{in}$ [39]	Decay constant of the IS weight	5 ms
$g_{max}$ [39]	Maximum value of the synaptic weight	0.015
$A_{+}$ [39]	Maximum correction value when the ES weight is increased	0.1
$A_{-}$ [39]	Minimum correction value when the ES weight is reduced	0.105
$B_{+}$ [39]	Maximum correction value when the IS weight is increased	0.02
$B_{-}$ [39]	Minimum correction value when the IS weight is reduced	0.03
$t_{+}$ [39]	Interval for the presynaptic and postsynaptic firing of neurons when the synaptic weights are increased	20 ms
$t_{-}$ [39]	Interval for the presynaptic and postsynaptic firing of neurons when the synaptic weights are reduced	20 ms

**Table 4:** Results for δ of SNNs with alternative topologies under stochastic attacks.

P (%)	5	10	15	20	25	30	35	40	45	50
Com-SNN	0.23	0.41	0.50	1.18	1.93	2.08	2.80	3.07	3.88	4.62
SF-SNN	0.48	1.23	1.95	2.11	2.92	2.95	3.85	3.64	4.28	5.81
SW-SNN	0.55	1.77	2.01	2.29	3.86	4.45	4.61	5.17	6.73	7.45

**Table 5:** Results for ρ of SNNs with alternative topologies under stochastic attacks.

P (%)	5	10	15	20	25	30	35	40	45	50
Com-SNN	0.997	0.996	0.993	0.991	0.985	0.981	0.972	0.966	0.957	0.955
SF-SNN	0.963	0.960	0.971	0.959	0.967	0.954	0.937	0.922	0.924	0.892
SW-SNN	0.965	0.962	0.975	0.955	0.960	0.945	0.923	0.895	0.856	0.834

**Table 6:** Relevance findings between progression of MSW and injury-resistance metrics.

P (%)	5	25	45
δ	0.931 **	0.907 **	0.924 **
ρ	−0.896 **	−0.920 **	−0.910 **

## Data Availability

The original contributions presented in this study are included in the article. Further inquiries can be directed to the corresponding author.
